# Evaluation of oral small molecule drugs for the treatment of COVID-19 patients: a systematic review and network meta-analysis

**DOI:** 10.1080/07853890.2023.2274511

**Published:** 2023-11-15

**Authors:** Zhaoyan Chen, Fangyuan Tian

**Affiliations:** a Department of Pharmacy, West China Hospital, Sichuan University, Chengdu, China; b Department of Epidemiology and Health Statistics, West China School of Public Health and West China Fourth Hospital, Sichuan University, Chengdu, China

**Keywords:** COVID-19, small molecule, randomized controlled trials, SARS-CoV-2, meta-analysis

## Abstract

**Introduction:**

At present, there are some randomized controlled trials (RCTs) of oral small molecule drugs. The purpose of this study was to evaluate the efficacy and safety of oral small molecule drug treatment for COVID-19.

**Methods:**

RCTs were identified through systematic searches of PubMed, Embase, and Cochrane Central Register of Controlled Trials through 1 April 2023. A total of nine RCTs were included, including 30,970 COVID-19 patients comparing five treatments (azvudine, molnupiravir, paxlovid, VV116, and placebo). The Cochrane risk of bias tool for randomized trials (RoB) was used to assess the bias risk of the included studies. The direct and indirect evidence were combined using a Bayesian network meta-analysis (PROSPERO Code No: CRD42023397837).

**Results:**

Direct analysis showed that paxlovid was associated with a reduced risk of mortality (odds ratio [OR] 0.12, 95% confidence interval [CI] 0.06–0.25) and hospitalization (OR = 0.04, 95% CI: 0.00–0.67) compared with placebo. Network meta-analysis showed that paxlovid had the highest probability of being the best management strategy in patients with COVID-19, reducing mortality (OR = 0.11, 95% CI: 0.01–1.99; surface under the cumulative ranking curve [SUCRA]: 0.77) and hospitalization (OR = 0.06, 95% CI: 0.00–1.03; SUCRA: 0.95). For prespecified safety outcomes, SUCRA values ranked VV116 (OR = 0.09, 95% CI: 0.00–2.07: SUCRA 0.86) as the most beneficial intervention for the prevention of serious adverse events.

**Conclusions:**

When compared to other antiviral medications, paxlovid can reduce the mortality and hospitalization of COVID-19 patients.

## 1. Introduction

Since December 2019, the coronavirus disease 2019 (COVID-19) epidemic has been reported in various parts of the world. To date, COVID-19 continues to spread globally and poses a threat to human health [1, 2]. As of 10 April 2023, the World Health Organization reported 762.2 million confirmed cases of COVID-19 worldwide, including 6.8 million fatalities [3]. One year after contracting COVID-19, only 29% of patients had totally recovered, and 71% had sequelae, according to UK research on more than 2000 COVID-19 inpatients. Fatigue, muscle soreness, bodily slowness, poor sleep, and dyspnoea are the most frequent side effects [4, 5]. According to a study on Chinese patients, sequelae such as weariness, muscle weakness, dyspnoea, and sleep issues still plagued 55% of inpatients 2 years after contracting COVID-19. The health status of COVID-19 patients was noticeably worse 2 years later than that of the overall population [6]. The risk of organ failure and mortality is greatly increased with each COVID-19 infection, according to certain research, and increases over time. These conditions include those that impact the kidneys, diabetes, heart, brain, blood, lungs, gastrointestinal tract, and musculoskeletal system, as well as mortality [7]. As a result, it is crucial to develop an efficient treatment strategy for COVID-19 patients [8] and to start using antiviral medications as soon as possible to address COVID-19-related symptoms. Doing so can help patients’ COVID-19 sequelae and lower their risk of hospitalization and mortality [9].

Remdesivir, chloroquine phosphate, and favipiravir are a few examples of broad-spectrum antiviral medications and medications targeting other viruses that were effective in combating the outbreak early on [10]. The oral small molecule drugs have had a significant impact on the prevention and management of the epidemic [11]. Oral drugs have unparalleled advantages. In contrast to most biotechnology products, which employ injectable drug delivery techniques and have low patient compliance, the majority of small molecule medications may be taken orally and are simple to administer. They are practical for therapy, including postinfection, preexposure, and postexposure treatments, and can be utilized in outpatient patients with minor symptoms. Small molecule medications have generally low sensitivity to storage settings, which facilitates storage and transportation. They can operate on intracellular and extracellular targets. Additionally, almost no immunogenicity exists, so allergic reactions may be minimal. Additionally, the targets are fairly developed, so even small changes could have unexpected results. Currently, 3CLpro (3C-like protease) and RdRp (RNA-dependent RNA polymerase) are the two main oral small molecule medicines being researched and developed for COVID-19 [12].

At present, the number of approved oral small molecule drugs for the treatment of COVID-19 for sale in China has increased to six, including two imported drugs, nirmatrelvir/ritonavir (paxlovid) and molnupiravir, as well as four domestic drugs, azvudine, remindevir (VV116), simnotrelvir/ritonavir (SIM0417), and leritrelvir (RAY1216). However, there is still a lack of evaluation of the efficacy and safety of different oral small molecule drugs in this group of patients. To gather more pertinent data for potential future clinical applications, we performed a network meta-analysis of randomized controlled trials (RCTs) to examine the efficacy and safety of several oral small molecule drugs.

## 2. Methods

### 2.1. Eligibility criteria

Studies were considered if they satisfied the following criteria: (1) looked at individuals with mild or moderate COVID-19; (2) used oral small molecule drug treatment (paxlovid, molnupiravir, azvudine, VV116, SIM0417, or RAY1216) as the experimental drug; (3) used oral small molecule drug, placebo or standard treatment as control; (4) the study was an RCT. Considering that there are relatively few RCT studies published on some drugs, in order to ensure the comprehensiveness of the studies, studies with smaller sample sizes are also included. Studies were not considered if they satisfied the following criteria: (1) the study was not an RCT or was a single-arm clinical trial; (2) unpublished RCTs with data available as preprints; (3) the studies conducted *in vitro*, abstracts from posters or conferences, and studies lacking adequate data for outcome analysis were disregarded.

### 2.2. Search strategy and literature screening

The literature was retrieved from PubMed, Embase, and Cochrane Central Register of Controlled Trials (CENTRAL). ‘COVID-19’, ‘SARS-CoV-2’, ‘paxlovid’, ‘molnupiravir’, ‘azvudine’, ‘VV116’, ‘SIM0417’, and ‘RAY1216’ were used to conduct a search from inception up to 1 April 2023. In order to determine whether further review was necessary, all titles and abstracts were evaluated. The first 50 references were separately evaluated for quality control by a senior researcher. The degree of agreement was 90%, with five inconsistencies that were discussed among the three reviewers to reach an agreement. Supplementary Appendix 1 provides a sample of the search strategy based on PubMed. To avoid overlooking any possibly pertinent studies, reference lists of selected papers were also examined. After using EndNote X7 to remove duplicates, the two researchers reviewed the titles and abstracts of these publications back-to-back for preliminary screening. They then examined the full text of any articles that initially satisfied the inclusion criteria to decide whether they were ultimately included. The third researcher oversaw reaching an agreement when the other two researchers could not. The International Prospective Register of Systematic Reviews received the protocol registration (PROSPERO Code No: CRD42023397837).

### 2.3. Data extraction

This systematic review and meta-analysis were reported using the preferred reporting items for systematic reviews and meta-analysis protocol (PRISMA-P) checklist guidelines. Each study that was included provided the following information, which was divided into three parts: (1) the fundamental details of the studies that were included, such as the initial author, the year of publication, the stage of clinical trials, the centre, the study population, the group information, the age range, and the sample size; (2) the effectiveness and safety of oral small molecule medications as measured by outcomes including death, hospitalization, adverse events, and significant adverse events; and (3) quality of the included studies assessed by the Cochrane Collaboration’s tool for assessing risk of bias [13].

### 2.4. Statistical analysis

We carried out Bayesian network meta-analyses with a consistency model in the R environment to encompass direct and indirect comparisons. For each treatment agent in relation to the reference treatment agent, the comparative safety and efficacy of any two treatment regimens were modelled. To combine the network results, we ran random effects models [14, 15]. After a burn-in of 5000 iterations, the models were based on 20,000 iterations. The odds ratio (OR) and 95% confidence intervals (CI) were used to compute the outcomes. The OR and 95% CI from direct comparisons are also presented. To determine the treatment agents’ ranking probabilities for each outcome, the surface under the cumulative ranking curve (SUCRA) was obtained [16]. The SUCRA numbers, which represent therapy rankings, range from 0% to 100%. A higher grade of therapy effectiveness corresponds to a larger area under the curve. Using the I2 statistic, the heterogeneity of treatment effects across the included trials was investigated. Low, moderate, and high heterogeneity are indicated by I2 values of 25%, 50%, and 75%, respectively. All analyses were performed in R software (version 4.2.0) and RevMan (version 5.4.0). A P value of <.05 was considered to indicate statistical significance.

## 3. Results

### 3.1. Study selection

A total of 1089 studies were found through the search of pertinent databases, including 261 from PubMed, 748 from Embase, and 80 from CENTRAL. A total of 928 studies were screened by the titles and abstracts, and 910 studies were eliminated after 161 duplicate studies had been eliminated. Eighteen studies were additionally excluded after reading the complete texts. The meta-analysis included 9 studies [17–25] in total (Supplementary Appendix 4; Table 1).

**Table 1.** Characteristics of the included published studies.

Article	Clinical trial registration	Phase	Centre	Study population	Intervention group	Control group	Age range	Sample size	
								Intervention group	Control group
Ren et al. [17]	ChiCTR2000029853	3	Single centre	Patients with mild and common COVID-19	Azvudine + standard treatment	Placebo + standard treatment	18 years and older	10	10
Fischer et al. [18]	NCT04405570	2a	Multicentre	Non-hospitalized with COVID-19	Molnupiravir, twice daily for 5 days	Placebo	18 years and older	140	62
Caraco et al. [19]	NCT04575597	3	Multicentre	Non-hospitalized with COVID-19	Molnupiravir, twice daily for 5 days	Placebo	18 years and older	228	74
Jayk Bernal et al. [20]	NCT04575597	3	Multicentre	Non-hospitalized adults with mild-to-moderate COVID-19	Molnupiravir, twice daily for 5 days	Placebo	18 years and older	716	717
Khoo et al. [21]	NCT04746183	2	Multicentre	Non-hospitalized adults with mild-to-moderate COVID-19	Molnupiravir, twice daily for 5 days	Placebo	18 years and older	90	90
Butler et al. [22]	ISRCTN30448031	NR	Multicentre	Patients with COVID-19	Molnupiravir, twice daily for 5 days	Usual care	18 years and older	12,774	12,934
Zou et al. [23]	ChiCTR2200056817	NR	Single centre	Patients with mild-to-moderate COVID-19	Molnupiravir, twice daily for 5 days	Usual care	Aged 18–80 years	77	31
Cao et al. [24]	NCT05341609	3	Multicentre	Patients with mild-to-moderate COVID-19	Paxlovid, twice daily for 5 days	VV116	18 years and older	387	384
Hammond et al. [25]	NCT04960202	2–3	Multicentre	Non-hospitalized with COVID-19	Paxlovid, twice daily for 5 days	Placebo	18 years and older	1120	1126

NR: not reported.

### 3.2. Study characteristics

A total of 30,970 participants from nine RCTs were used in the study, and 15,926 of these patients were assigned to different treatment plans. All studies have disclosed protocols on the clinical trial registration website, of which seven are multicentre studies. Patients with mild or moderate COVID-19 made up the majority of the study population. Finally, three oral small molecule drugs were included in this study. Among them, the most reported was molnupiravir, with six RCTs. Two studies reported that patients received paxlovid, and one study reported azvudine in the intervention group. The quality of the included studies is shown in Supplementary Appendix 2. The study’s overall calibre is quite good. Details of these RCTs are shown in Table 1.

### 3.3. Efficacy of small molecule drugs

**Figure 1.**
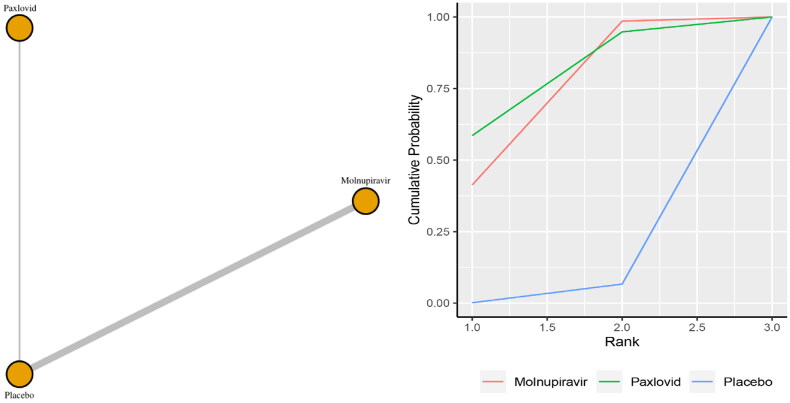
(a) Network plot of all-cause mortality; (b) SUCRA-based ranking probability graph of each medication.

Five studies reported the incidence of mortality. The network plot for comparisons between the different management strategies for mortality is presented in Figure 1a. According to the direct analysis, paxlovid (OR = 0.12, 95% CI: 0.06–0.25; Supplementary Appendix 5) and molnupiravir (OR = 0.25, 95% CI: 0.09–0.68; Supplementary Appendix 6) showed better efficacy in reducing mortality than placebo or standard care. There was a significant difference between the paxlovid or molnupiravir group and the control group. Network analysis showed that the pooled OR was 0.11 (95% CI: 0.01–1.99) for mortality comparing paxlovid with placebo, and the pooled OR was 0.15 (95% CI: 0.01–0.82) for mortality comparing molnupiravir with usual care or placebo (Supplementary Appendix 3). The summary OR, however, failed to show any statistically significant distinctions between paxlovid and placebo. The molnupiravir group, however, significantly differed from the usual care or placebo group. With a SUCRA value of 0.77, paxlovid had the highest likelihood of being the best management approach for COVID-19 patients who experienced mortality (Figure 1b).

**Figure 2.**
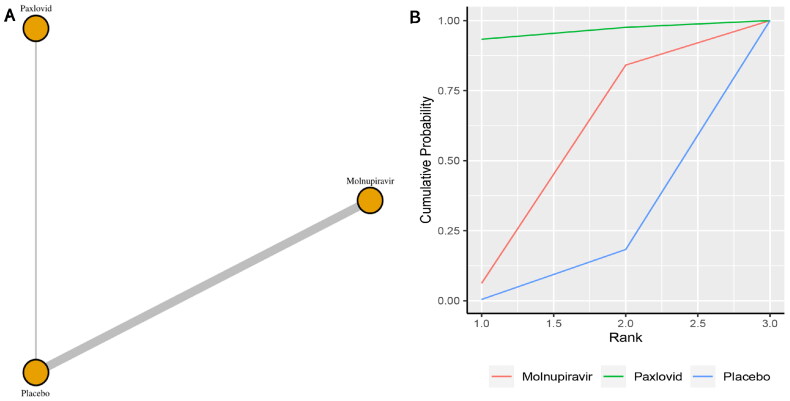
(a) Network plot of all-cause hospitalization; (b) SUCRA-based ranking probability graph of each medication.

Six studies reported the incidence of hospitalization. The network plot for comparisons between the different management strategies for hospitalization is presented in Figure 2a. According to the direct analysis, paxlovid (OR = 0.04, 95% CI: 0.00–0.67; Supplementary Appendix 6) showed better efficacy in reducing mortality than placebo. There was a significant difference between the paxlovid and placebo groups. However, there was no significant difference between the molnupiravir group and the usual care or placebo group (OR = 0.91, 95% CI: 0.73–1.14; Supplementary Appendix 6). Network analysis showed that the pooled OR was 0.06 (95% CI: 0.00–1.03) for hospitalization comparing paxlovid with placebo, and the pooled OR was 0.68 (95% CI: 0.15–1.68) for hospitalization comparing molnupiravir with usual care or placebo (Supplementary Appendix 3). There were no statistically significant differences between the two medications and the control group, according to the summary OR. With a SUCRA score of 0.95, paxlovid had the highest likelihood of being the best care option for patients with COVID-19 for the outcome of hospitalization (Figure 2b).

### 3.4. Safety of small molecule drugs

**Figure 3.**
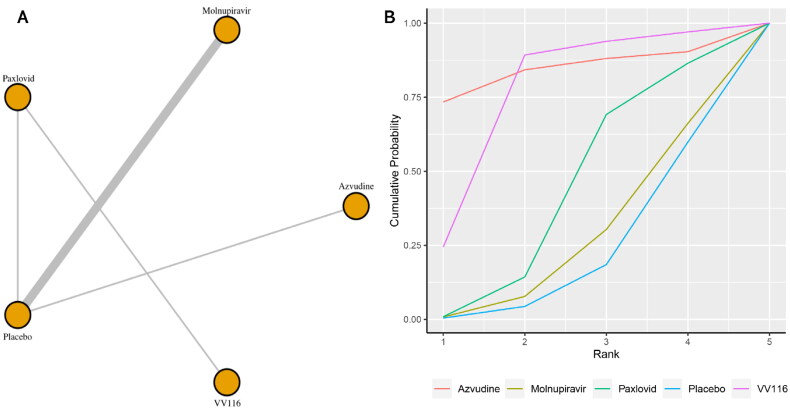
(a) Network plot of all-cause adverse events; (b) SUCRA-based ranking probability graph of each medication.

Eight studies reported the incidence of adverse events. Figure 3a shows the network plot for comparing the various management approaches for adverse occurrences. According to the direct analysis, VV116 showed better efficacy in reducing adverse events than paxlovid (OR = 1.64, 95% CI: 1.19–2.26; Supplementary Appendix 7). There was no significant difference between the azvudine group (OR = 0.10, 95% CI: 0.00–2.28; Supplementary Appendix 7), molnupiravir group (OR = 0.94, 95% CI: 0.77–1.13; Supplementary Appendix 7), paxlovid group (OR = 0.85, 95% CI: 0.72–1.00; Supplementary Appendix 5) and the usual care or placebo group. Network analysis revealed that there were no statistically significant differences between the groups taking these medicines according to the summary OR. Azvudine, with a SUCRA value of 0.85, had the highest likelihood of being the best management plan for the outcome of adverse events in patients with COVID-19 (Figure 3b). Azvudine (SUCRA: 0.76) and paxlovid (SUCRA: 0.43) ranked as the second and third most beneficial interventions for this outcome.

**Figure 4.**
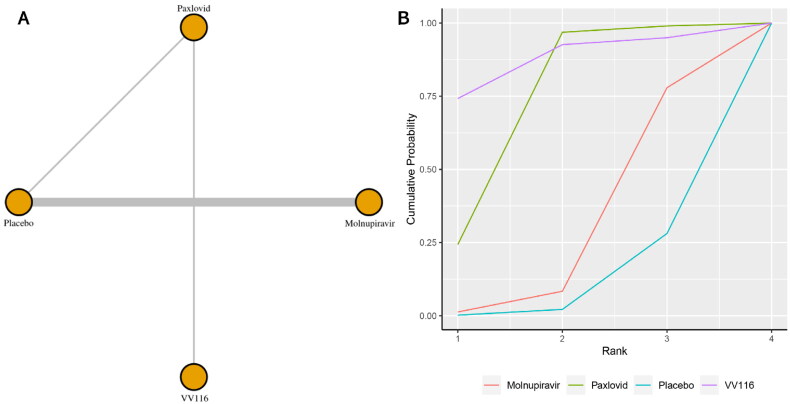
(a) Network plot of all-cause serious adverse events; (b) SUCRA-based ranking probability graph of each medication.

Seven studies reported the incidence of serious adverse events. Figure 4a shows the network plot for comparing the various management approaches for significant adverse events. According to the direct analysis, paxlovid (OR = 0.23, 95% CI: 0.14–0.39; Supplementary Appendix 8) showed better efficacy in reducing serious adverse events than placebo. There was no significant difference between the molnupiravir group (OR = 0.86, 95% CI: 0.66–1.13; Supplementary Appendix 8) and the usual care or placebo group. Network analysis showed that the pooled OR was 0.23 (95% CI: 0.06–0.78) for serious adverse events comparing paxlovid with placebo (Supplementary Appendix 3). There was a significant difference between the paxlovid group and the placebo group. With a SUCRA value of 0.86, VV116 had the highest likelihood of being the best care plan for patients with COVID-19 for the outcome of significant adverse events (Figure 4b). The second and third most effective therapies for this outcome were paxlovid (SUCRA: 0.74) and molnupiravir (SUCRA: 0.29), respectively.

## 4. Discussion

A total of 19.8 million fatalities were avoided globally in the first year of the COVID-19 vaccine (from 8 December 2020 to 8 December 2021) [26], suggesting that vaccination may be the best method of managing COVID-19. The COVID-19 vaccine drastically changed the course of the pandemic and helped tens of millions of people around the world. However, due to the difficulty with public acceptance of vaccines, particularly for older groups, public immunization takes some time [27]. The COVID-19 outbreak primarily impacted the elderly population [28]. The mutant strain of omicron, which has a fast transmission rate, a high transmission intensity, and a hidden transmission procedure, is also the dominant strain in the pandemic right now [29]. In contrast to certain prior COVID-19 viruses, once there is an infectious source, infection risk continues to rise even after receiving the COVID-19 vaccination [30]. It is crucial to develop an efficient treatment strategy for COVID-19 patients and to start using antiviral medications as soon as possible to address COVID-19-related symptoms.

On 12 February 2022, Paxlovid received approval from China’s National Medical Products Administration (NMPA) for an urgent evaluation. On 14 March 2022, the People’s Republic of China’s National Health Commission published the Scheme for Diagnosis and Treatment of SARS-CoV-2 (The 9th Trial Edition). Paxlovid was initially listed as an antiviral medication in the diagnosis and treatment plan [31]. Azvudine was officially launched on 25 July 2022, becoming the second oral small molecule drug for COVID-19 in mainland China after paxlovid. Molnupiravir was approved by the NMPA of China for emergency review on 30 December 2022. It was officially launched on 13 January 2022, becoming the third small molecule oral drug for COVID-19 in mainland China after paxlovid and azvudine. The National Health Commission of the People’s Republic of China released the Scheme for Diagnosis and Treatment of SARS-CoV-2 (The 10th Trial Edition) on 6 January 2023 [32]. Compared with the 9th Edition of the diagnosis and treatment plan previously released, in addition to paxlovid, monoclonal antibody, intravenous injection of human immunoglobulin and convalescent plasma, the antiviral treatment content added azvudine and molnupiravir [33]. Later, VV116, SIM0417, and RAY1216 domestic drugs were launched in mainland China to treat COVID-19.

This study included nine RCTs, involving three types of drugs, including six studies on molnupiravir, two studies on paxlovid, and one study on azvudine. The research population mainly revolves around mild-to-model COVID-19 patients. Most studies are conducted in multiple centres. Effectiveness is a crucial metric for oral small molecule medication therapy for COVID-19. Paxlovid can considerably lower the percentage of patients who die and end up in hospitals. Paxlovid had the highest likelihood of being the best efficacy management option in patients with COVID-19 when compared to molnupiravir and placebo. This demonstrates that paxlovid is more effective at lowering the efficacy outcomes of COVID-19. Similar research has shown that molnupiravir and paxlovid can both reduce the incidence of severe COVID-19, which is defined as hospitalization or mortality for high-risk patients, by approximately 30% and over 90%, respectively [34]. A direct comparison of antivirals showed that paxlovid was superior in lowering long-term hospitalization and mortality, even if the other antivirals similarly had a decreased risk of invasive mechanical ventilation [32, 35].

There was no difference in the number of adverse events or series of adverse events between the paxlovid and molnupiravir groups, indicating that the safety of these two medications was similar. Compared with paxlovid and molnupiravir, VV166 and azvudine had a greater probability of being the best safety management strategy in patients with COVID-19. Nirmatrelvir and ritonavir, which comprise paxlovid and are both CYP3A substrates, are metabolized differently by different medications, which alters the efficacy and safety of nirmatrelvir and ritonavir. Nirmatrelvir alone is a potent CYP3A irreversible inhibitor that can raise the blood concentration of other CYP3A substrates, improving the effectiveness of combination medications or raising the risk of negative side effects [36]. Some researchers have raised persistent concerns about the RNA mutation ability of molnupiravir; that is, it will cause mutations in the patient’s own genetic material, which may lead to cancer or congenital defects [37]. This needs further exploration in follow-up research.

This study’s key strength is that it is the first, to the best of our knowledge, network meta-analysis on the efficacy and safety of oral small molecule antiviral drug treatment in patients with COVID-19. To lessen any bias, this article underwent thorough quality evaluation procedures. Only published RCTs were included for a thorough analysis in this meta-analysis, which also used strict and practical inclusion criteria. However, this study also has some limitations. First, there are few studies available on some medications that may be examined, such as paxlovid and azvudine. There is a significant disparity in the sample size, outcome indicators, and population of RCTs in the study, and there is a paucity of information regarding the effectiveness and safety of SIM0417 and RAY1216 on COVID-19 patients in clinical practice. Additionally, this article was unable to conduct subgroup analysis based on risk factors such as older age and vaccination status due to a lack of data, which may have impacted the accuracy of the findings. Therefore, more extensive and high-quality studies are still needed to confirm the effectiveness and safety of oral small molecule drug treatment for COVID-19.

## 5. Conclusion

When compared to other antiviral medications, paxlovid can lower the mortality and hospitalization of COVID-19 patients. According to the safety results, VV166 has good safety. More extensive research is needed to confirm these conclusions.

## Supplementary Material

Supplemental Material
