# Supplementary material for: Evaluation of oral small molecule drugs for the treatment of COVID-19 patients: a systematic review and network meta-analysis
Source: Ann Med. 2023 Nov 15;55(2):2274511. doi: 10.1080/07853890.2023.2274511 (PMC10768866; doi:10.1080/07853890.2023.2274511)
Supplement: Supplemental Material [file IANN_A_2274511_SM4050.docx]

**Evaluation of oral small molecule drugs for the treatment of COVID-19 patients: a systematic review and network meta-analysis**

Contents:

Appendix 1. A sample of the search strategy based on PubMed

Appendix 2. Quality assessment of included studies

Appendix 3. Network meta-analyses of the efficacy and safety of oral small-molecule antiviral drugs for COVID-19 patients

Appendix 4. Flow chart of study selection

Appendix 5. Forest plot of mortality in the direct meta-analysis

Appendix 6. Forest plot of hospitalization in the direct meta-analysis

Appendix 7. Forest plot of adverse events in the direct meta-analysis

Appendix 8. Forest plot of serious adverse events in the direct meta-analysis

Appendix 1. A sample of the search strategy based on PubMed

| 1 | " **covid 19**"[MeSH] |
| --- | --- |
| 2 | " **sars cov 2 infection** "[title/abstract] |
| 3 | " **covid 19***"[title/abstract] |
| 4 | "**2019 ncov infection** "[title/abstract] |
| 5 | " **SARS-CoV-2**"[title/abstract] |
| 6 | “**novel coronavirus 2019**”[title/abstract] |
| 7 | " **COVID-19**"[title/abstract] |
| 8 | 1 or 2 or 3 or 4 or 5 or 6 or 7 |
| 9 | "**Paxlovid**"[title/abstract] |
| 10 | “**Nirmatrelvir/Ritonavir**“[title/abstract] |
| 11 | “**Nirmatrelvir**”[title/abstract] |
| 12 | 9 or 10 or 11 |
| 13 | **“molnupiravir”**[title/abstract] |
| 14 | **“EIDD-2801“**[title/abstract] |
| 15 | **“MK-4482”**[title/abstract] |
| 16 | 13 or 14 or 15 |
| 17 | "azvudine"[ title/abstract] |
| 18 | “fnc“[title/abstract] |
| 19 | 17 or 18 |
| 20 | “Remindevir”[title/abstract] |
| 21 | “VV116”[title/abstract] |
| 22 | 20 or 21 |
| 23 | “Simnotrelvir/Ritonavir” [title/abstract] |
| 24 | “SIM0417” [title/abstract] |
| 25 | 23 or 24 |
| 26 | “Leritrelvir” [title/abstract] |
| 27 | “RAY1216” [title/abstract] |
| 28 | 26 or 27 |
| 29 | **“randomized controlled trial”**[pt] |
| 30 | **“controlled clinical trial”**[pt] |
| 31 | **“randomized“**[Title/Abstract] |
| 32 | **“placebo“**[Title/Abstract] |
| 33 | **“clinical trials as topic“**[**mesh:noexp**] |
| 34 | **“randomly”**[Title/Abstract] |
| 35 | **“trial”**[Title] |
| 36 | **“animals”**[mh] |
| 37 | **“humans”**[mh] |
| 38 | **“animals”**[mh] |
| 39 | 29 or 30 or 31 or 32 or 33 or 34 or 35 not (36 not (37 and 38)) |
| 40 | 12 or 16 or 19 or 22 or 25 or 28 |
| 41 | 8 and 39 or 40 |

Appendix 2. Quality assessment of included studies

| study | Selection bias | | Performance bias | Detection bias | Attrition bias | Reporting bias |  |  |  |
| --- | --- | --- | --- | --- | --- | --- | --- | --- | --- |
|  | Random sequence generation | Allocation concealment | Blinding of participants and personnel | Blinding of outcome assessment | Incomplete outcome data | Selective reporting | Other bias | Total score (max =7) |  |
|  |  |  |  |  |  |  |  |  |  |
| Ren, et al. 2020 | + | + | + | + | + | + | + | 7 |  |
| Fischer, et al. 2022 | + | + | + | + | + | + | + | 7 |  |
| Caraco, et al. 2021 | + | + | + | + | + | + | + | 7 |  |
| Jayk, et al. 2022 | + | + | + | + | + | + | + | 7 |  |
| Khoo, et al. 2022 | + | + | + | + | + | + | + | 7 |  |
| Butler, et al. 2022 | + | + | + | + | + | + | + | 7 |  |
| Zou, et al. 2022 | + | ? | ? | ? | + | + | ? | 3 |  |
| Cao, et al. 2023 | + | + | + | + | + | + | + | 7 |  |
| Hammond, et al. 2022 | + | + | + | + | + | + | + | 7 |  |

Appendix 3. Network meta-analyses of the efficacy and safety of oral small-molecule antiviral drugs for COVID-19 patients

| **Treatment** | **odds ratio (OR)** | | | |
| --- | --- | --- | --- | --- |
|  | **Mortality** | **Hospitalization** | **Adverse events** | **Serious adverse event** |
| Molnupiravir vs. Placebo | **0.15 (0.01, 0.82)*** | 0.68 (0.15, 1.68) | 0.98 (0.68, 1.68) | 0.84 (0.4, 1.56) |
| Molnupiravir vs. Paxlovid | 1.36 (0.02, 30.47) | 11.61 (0.38, 494.54) | 1.15 (0.51, 3.14) | 3.66 (0.86, 14.78) |
| Paxlovid vs. Placebo | 0.11 (0.01, 1.99) | 0.06 (0, 1.03) | 0.85 (0.38, 1.86) | **0.23 (0.06, 0.78)*** |
| VV116 vs. Molnupiravir | NA | NA | 0.53 (0.14, 1.65) | 0.11 (0, 2.69) |
| VV116 vs. Paxlovid | NA | NA | 0.61 (0.27, 1.39) | 0.39 (0.01, 7.07) |
| VV116 vs. Placebo | NA | NA | 0.51 (0.17, 1.62) | 0.09 (0, 2.07) |
| Azvudine vs. Molnupiravir | NA | NA | 0.19 (0.01, 2.51) | NA |
| Azvudine vs. Paxlovid | NA | NA | 0.22 (0.01, 3.18) | NA |
| Azvudine vs. VV116 | NA | NA | 0.36 (0.01, 5.9) | NA |
| Azvudine vs. Placebo | NA | NA | 0.19 (0.01, 2.42) | NA |
|  |  |  |  |  |
| NA: Not available, *Statistically significant (P＜0.05) | | | | |

Appendix 4. Flow chart of study selection


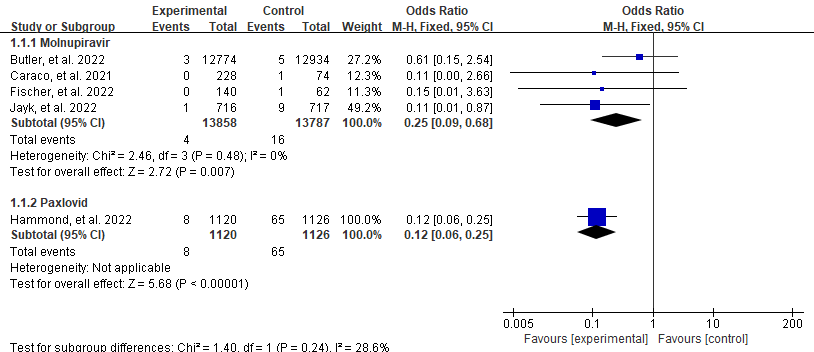


Appendix 5. Forest plot of mortality in the direct meta-analysis


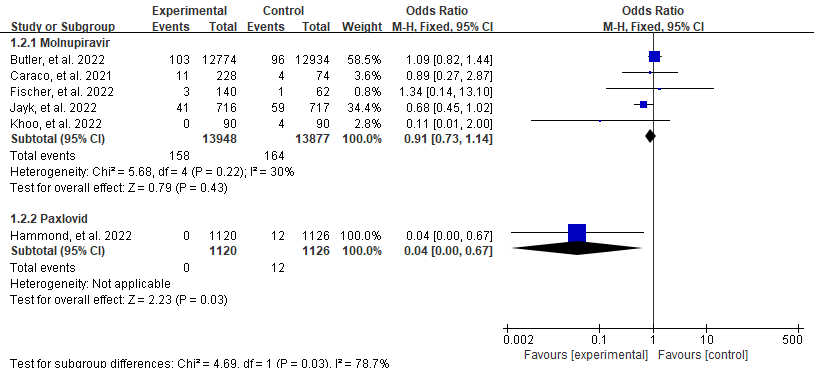


Appendix 6. Forest plot of hospitalization in the direct meta-analysis


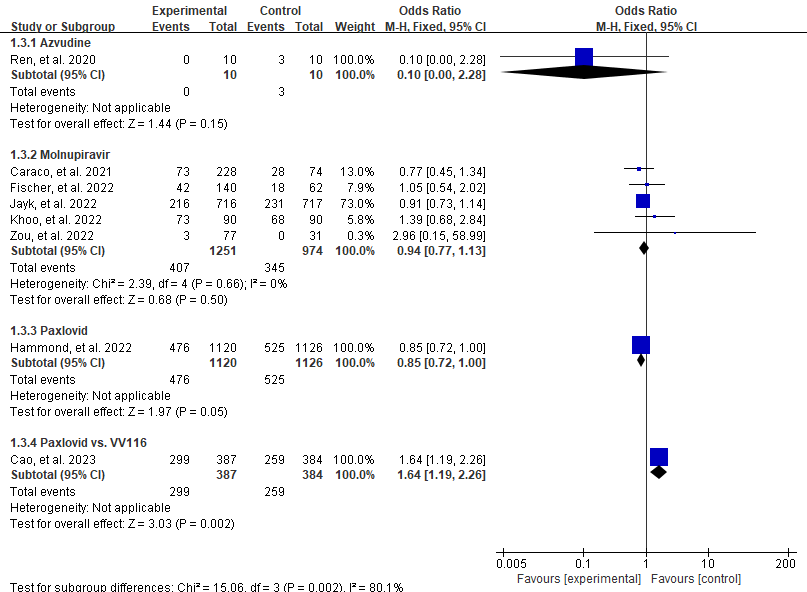


Appendix 7. Forest plot of adverse events in the direct meta-analysis


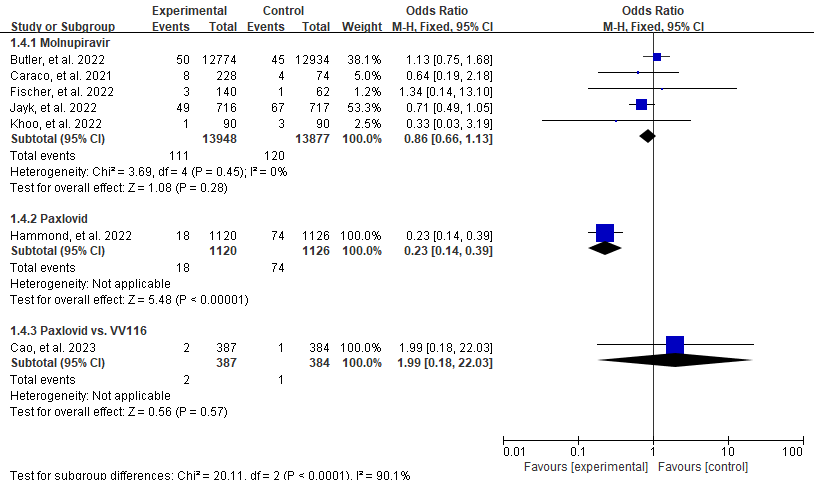


Appendix 8. Forest plot of serious adverse events in the direct meta-analysis
